# Effects of Recreational Boating on Microbial and Meiofauna Diversity in Coastal Shallow Ecosystems of the Baltic Sea

**DOI:** 10.1128/mSphere.00127-21

**Published:** 2021-09-01

**Authors:** Sven Iburg, Dandan Izabel-Shen, Åsa N. Austin, Joakim P. Hansen, Johan S. Eklöf, Francisco J. A. Nascimento

**Affiliations:** a Department of Ecology, Environment and Plant Sciences (DEEP), Stockholm University, Stockholm, Sweden; b Stockholm University Baltic Sea Centre, Stockholm, Sweden; University of Wisconsin—Madison

**Keywords:** Baltic Sea, benthic community composition, coastal ecosystems, macrophytes, microbial ecology, recreational boating, shoreline development

## Abstract

Recreational boating can impact benthic ecosystems in coastal waters. Reduced height and cover of aquatic vegetation in shallow Baltic Sea inlets with high boat traffic have raised concerns about cascading effects on benthic communities in these ecosystems. Here, we characterized the diversity and composition of sediment-associated microbial and meiofaunal communities across five bays subjected to low and high degrees of boating activity and examined the community-environment relationships and association with bay morphometry. We found that recreational boating activity altered meiofauna alpha diversity and the composition of both micro- and meiobenthic communities, and there were strong correlations between community structure and morphometric variables like topographic openness, wave exposure, water surface area, and total phosphorous concentrations. Inlets with high boat traffic showed an increase of bacterial taxa like *Hydrogenophilaceae* and *Burkholderiaceae*. Several meiofauna taxa previously reported to respond positively to high levels of suspended organic matter were found in higher relative abundances in the bays with high boat traffic. Overall, our results show that morphometric characteristics of inlets are the strongest drivers of benthic diversity in shallow coastal environments. However, while the effects were small, we found significant effects of recreational boating on benthic community structure that should be considered when evaluating the new mooring projects.

**IMPORTANCE** With the increase of recreational boating activity and development of boating infrastructure in shallow, wave-protected areas, there is growing concern for their impact on coastal ecosystems. In order to properly assess the effects and consider the potential for recovery, it is important to investigate microbial and meiofaunal communities that underpin the functioning of these ecosystems. Here, we present the first study that uses DNA metabarcoding to assess how benthic biodiversity in shallow coastal areas is impacted by recreational boating. Our study shows a relatively small, but significant, effect of recreational boating both on meiofauna alpha diversity and meiofauna and bacterial community composition. However, both meiofauna and bacterial community composition in shallow benthic habitats is mediated to a higher degree by abiotic variables, such as topographic openness, area or size of the inlets, and wave exposure. Despite the fact that the effects were small, such impacts on benthic biodiversity should be considered in the management of coastal shallow habitats.

## INTRODUCTION

Coastal ecosystems have received substantial attention in the field of aquatic ecology, as these habitats are subjected to progressively increased anthropogenic disturbances (1, 2). They serve as important ecological buffers between the distinct terrestrial and deeper marine environments through, e.g., flow-driven sedimentation and the exchange of salt water and freshwater (3, 4). Human activities such as land use and coastal development result in the runoff of pollutants and nutrients into coastal waters (reference 5 and references therein) and thereby increase hypoxia and sediment flux in benthic habitats (6). In comparison with open waters, soft sediment bottoms in coastal marine environments are greatly impacted by multiple anthropogenic stressors in terms of habitat degradation and biodiversity loss (7, 8). A growing body of literature shows a decrease in macrophytes, macroinvertebrates, and fish communities in the degraded habitats (such as low resilience and reduced ecosystem services) (9–11).

The effects of large-scale disturbances such as eutrophication and fishing on coastal ecosystems have become a major focus of research during the last decades (12–14). However, small-scale disturbances induced by shoreline development and recreational boat traffic are emerging but considerably less studied concerns (15). Given the slow recovery of some benthic organisms from disturbance (16, 17), press disturbances (long-term disturbances that have long-term effects on an ecosystem) from shoreline construction and recreating boating may exert extensive and long-lasting effects on sediment habitats. A number of studies have shown both a reduction in species richness and altered composition of aquatic macrophytes in shallow inlets due to intense recreational boat activities associated with expansion of infrastructure (10, 18, 19). For example, changes in diversity and composition of macrophytes, together with abiotic factors such as boat-generated waves and dredge spoil disposal, have been shown to alter assemblages of fish and macroinvertebrates (19–21). From a broad ecological perspective, the direct effect of recreational boating on aquatic vegetation can result in a cascading effect on biodiversity (22), stability of physiochemical variables such as water turbidity (23), and organic matter content (24). It is clear that maritime traffic imposes a series of environmental challenges or disturbances with a wide range of consequences for marine life (5).

Microbial communities drive biogeochemical processes, sustain the bases of food webs, and recycle elementary nutrients within every ecosystem (25). Because of their fundamental roles in driving important ecosystem processes, understanding how microbial communities respond to press disturbance can provide insights into the potential for ecosystems to recover. However, to the best of our knowledge, no existing studies have assessed the ecological consequences of boating-induced disturbances on microbial and microeukaryotic communities. With the damage to aquatic vegetation from mechanical shearing, boat hulls, anchors, and sediment disturbance, the rhizosphere in these coastal ecosystems is likely to be affected. Rhizosphere communities are known to underpin crucial geochemical processes in sediment layers like sulfide oxidation and nitrate reduction (26, 27) as they are ideal habitats for important microbial taxa like cable bacteria (28). Moreover, as aquatic vegetation helps stabilize the sediment structure and supplies the rhizosphere with oxygen in the deeper layers of the sediment (29), the reduction of, and damage to, aquatic macrophytes is likely to affect these root-associated microbial communities and large microeukaryotes that feed or depend on these interactions. A long-term field experiment demonstrated that disturbance of seagrass meadows in coastal ecosystems led to substantial changes in meiobenthic community composition, especially the trophic structure of the nematode community (30). Given the known effects of boat-induced disturbances on aquatic vegetation and the potential of cascading effects, a better understanding of microbial and meiofaunal responses to press disturbances is necessary to move toward the goal of biodiversity monitoring managements using bioindicator species (31, 32).

**FIG 1:**
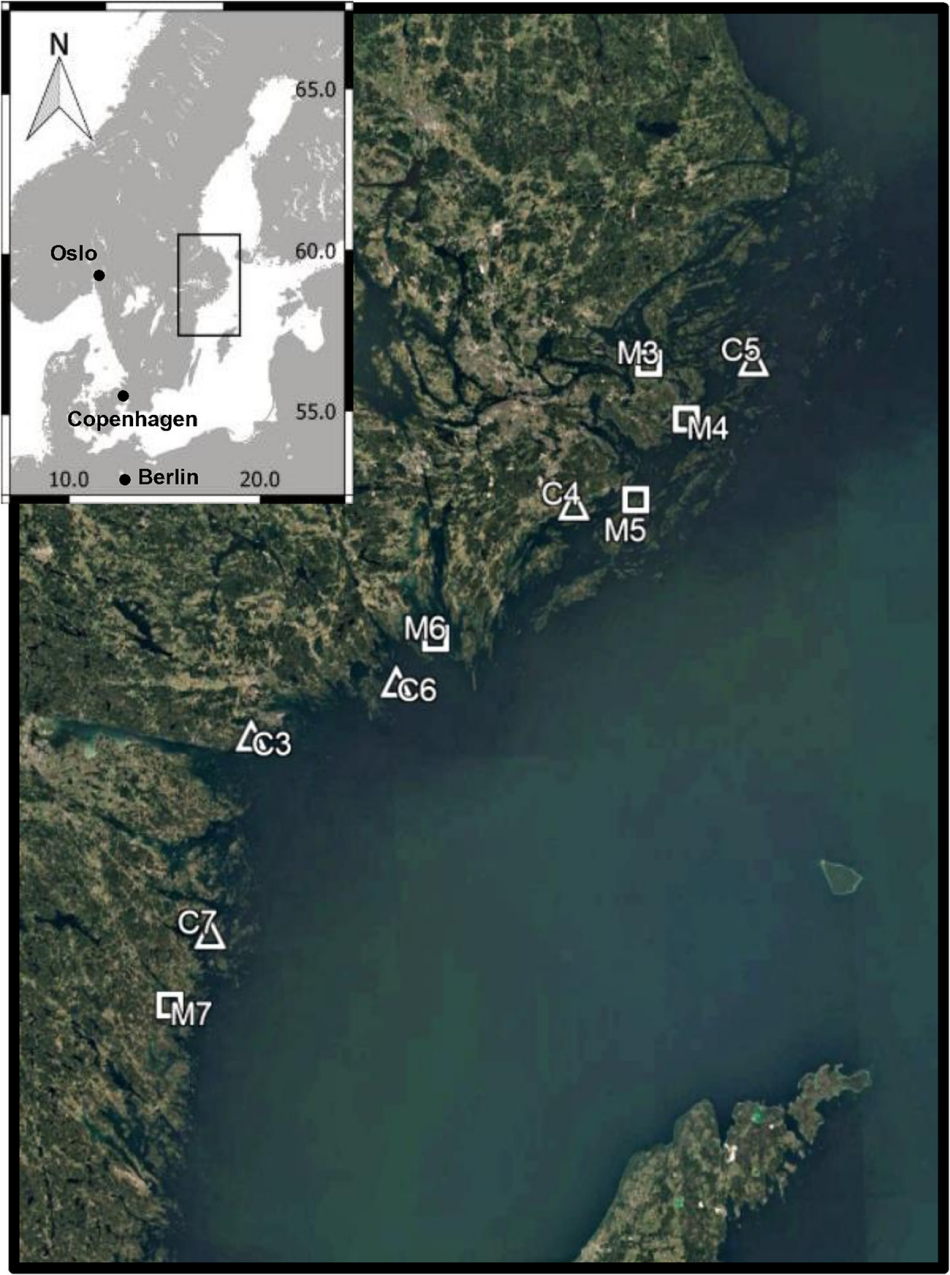
Map of the Baltic Sea coastline. The locations of the 10 shallow bays are denoted with squares (marina) and triangles (control). The map was created using QGIS v. 2.12.3 (37) and Google Earth Pro v 7.3.2. The bay codes, together with the mooring berths per hectare in each bay, are listed in Table 2.

In this study, we aimed to assess the responses of microbial (i.e., bacteria) and meiofaunal communities (i.e., benthic microscopic animals smaller than 1 mm, but retained in 0.04-mm sieve) to recreational boating using a field survey in shallow coastal bays in the Baltic Sea. The Baltic Sea contains a high variety of coastal regions differing in their geomorphology (33, 34), and shallow waters of most regions are covered by submerged macrophytes. With the increase of recreational boating activity and development of boating infrastructure along the Baltic Sea coast, particularly in shallow, wave-protected areas (35), there is growing concern for their impact on the coastal ecosystems. Recent work has evidenced negative effects of recreational boating in the Baltic Sea on coastal vegetation and associated fish assemblages (10, 19, 36). A previous study using data from the same field survey found that vegetation cover and height were lower in marinas (bays with a high level of boating) than control bays (bays with a low level of boating), with potential knock-on effects on associated fish assemblages (10). Here, we advance this work through high-throughput sequencing of the prokaryotic 16S and eukaryotic 18S ribosomal subunit rRNA genes to characterize the diversity and composition of meiofauna and microbial communities along the bays with different intensity of boat activities (Fig. 1) and identify the main environmental drivers of benthic diversity in shallow coastal areas. We hypothesized the following. (i) Alpha diversity of meiofauna and bacteria is lower in bays exposed to a high intensity of recreational boating traffic than in control bays with no or very low intensity of boating. (ii) Bacterial and meiofauna community structure in bays with high recreational boat traffic differs from that in control areas. (iii) Given the relatively fast proliferation and high dispersal rate of bacteria compared to (larger) microeukaryotes (37), they can rapidly colonize the open niches upon disturbances by boating. Thus, bacteria are likely less affected by boating than the meiofauna communities.

## RESULTS

### Amplicon sequencing output.

After trimming and filtering, 11,402,274 reads were obtained for the 16S rRNA data set and 11,087,582 reads for the 18S rRNA data set, with an average sequence depth of 186,922 for 16S (range, 49,160 to 838,283) and 186,995 reads per sample for 18S (110,618 to 346,142). Taxonomy was assigned to 91,014 amplicon sequence variants (ASVs) from the 16S data set using the SILVA database as a reference. After filtering out singletons, 67,330 ASVs remained. The 18S data set yielded 25,003 ASVs assigned through the BLASTn database as described in previous studies (38, 39). We found that at the phylum level, the majority of bacterial ASVs were affiliated with *Proteobacteria* (average ± standard deviation, 50.63% ± 3.46%), *Actinobacteria* (13.65% ± 0.84%), *Bacteroidetes* (9.22% ± 0.81%), and *Acidobacteria* (3.35% ± 0.22%). For the 18S data set, we subset ASVs assigned to eukaryote and metazoan taxa. Across all samples, the majority of metazoan ASVs were affiliated with to Arthropoda (65.61% ± 19.75%), Nematoda (10.89% ± 2.84%), and Mollusca (Gastropod and Bivalvia; 6.09% ± 1.23%).

### Alpha diversity.

**FIG 2:**
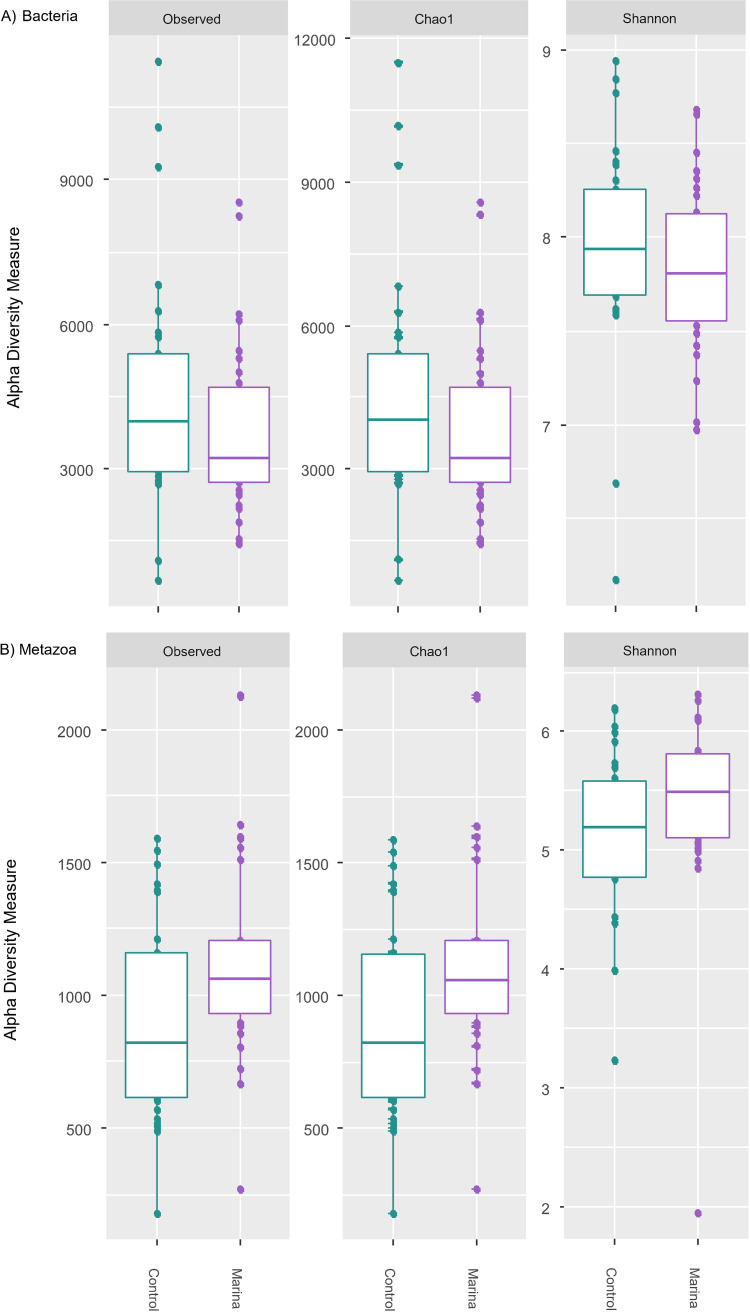
Alpha diversity by bay type (observed richness, Chao1, and Shannon) for control and marina bays for bacteria (A) and metazoa (B).

Alpha diversity indices for bacterial communities did not differ between control and marina bays (analysis of variance [ANOVA], *P* = 0.16, Fig. 2A; see also Table S2 in the supplemental material). In contrast, both Shannon and Chao1 alpha diversity estimates for metazoans, but not for observed number of ASVs, were significantly higher in marinas than in controls (generalized linear mixed model [GLMM], *P* = 0.034, 0.007, and 0.87, respectively, Fig. 2B; see Table S3 in supplemental material for statistical details).

10.1128/mSphere.00127-21.5 TABLE S2 Statistical analysis for GLMM used to assess the effects of marinas of bacterial alpha diversity. Type, marina versus control Table S2, CSV file, 0.001 MB. Copyright © 2021 Iburg et al. 2021 Iburg et al. https://creativecommons.org/licenses/by/4.0/ This content is distributed under the terms of the Creative Commons Attribution 4.0 International license.

10.1128/mSphere.00127-21.6 TABLE S3 Statistical analysis for GLMM used to assess the effects of marinas of metazoan alpha diversity. Type, marina versus control Table S3, CSV file, 0.002 MB. Copyright © 2021 Iburg et al. 2021 Iburg et al. https://creativecommons.org/licenses/by/4.0/ This content is distributed under the terms of the Creative Commons Attribution 4.0 International license.

### Community structure.

**FIG 3:**
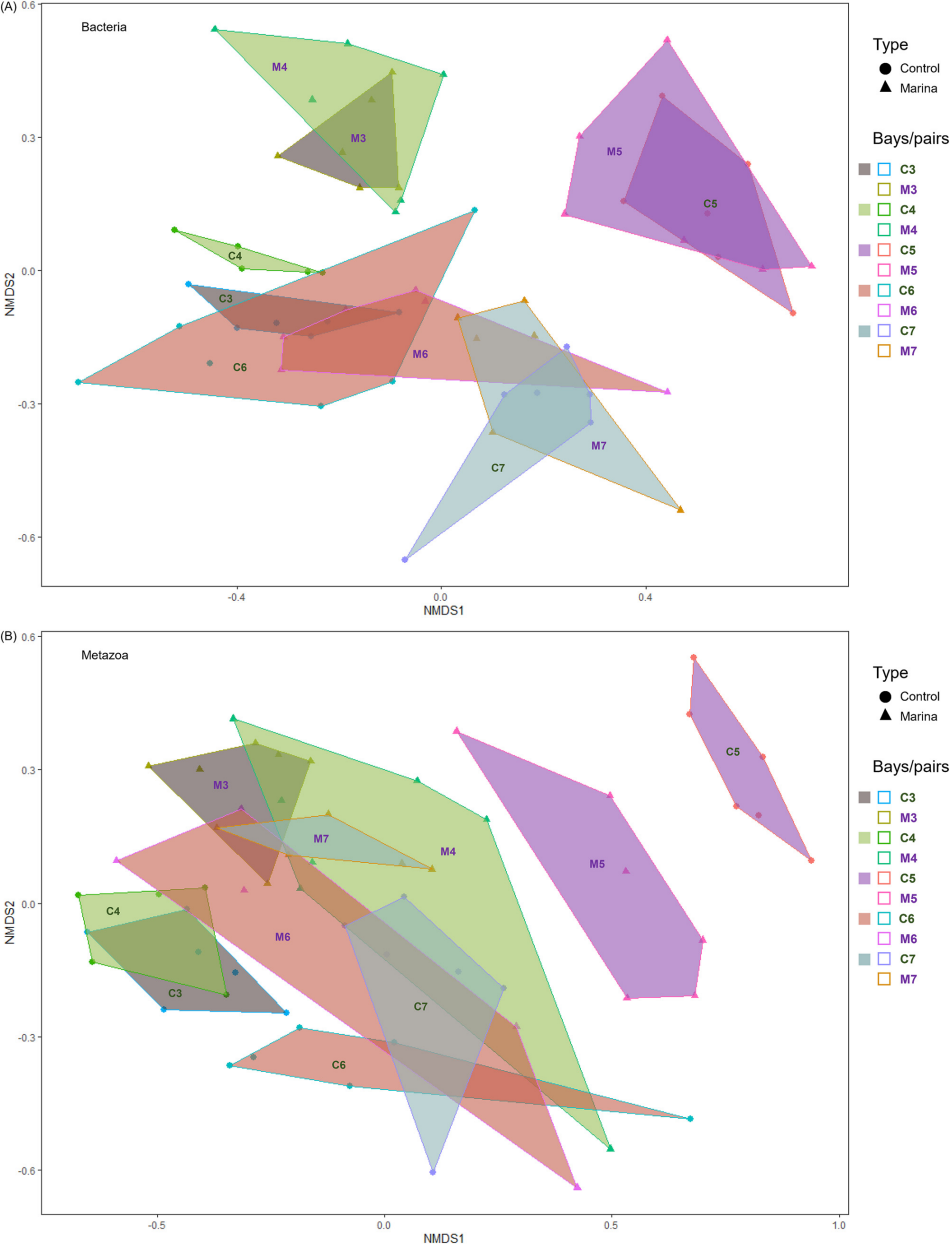
NMDS of Bray-Curtis dissimilarities based on the relative abundance of all ASVs classified as bacteria (stress = 0.18) (A) and metazoa (stress = 0.019) (B). Each data point represents a replicate in individual bays. Colored polygons cluster the samples collected from paired bays. Point shapes and lines depict bay types and are labeled with suffixes (marina [M] and control [C]).

Differences in bacterial and meiofauna community structure between marina and control bays were minor, but statistically significant (Fig. 3A and B, respectively; *adonis*, permutational multivariate analysis of variance [PERMANOVA], *P* < 0.001 in both tests; Table S3). Bacterial communities were clustered strongly by bay pairs compared to bay type (marinas versus control). The bacterial community structure in three of the five paired bays overlapped with the C3/M3 and C4/M4 pairs being clearly distinct. Again, while the difference between marina and control bays was minor, it was significant (PERMANOVA, *P* = 0.001, *R*^2^ = 0.036 [Table S5]). The nonmetric multidimensional scaling (NMDS) plot on metazoan analysis (Fig. 3B) revealed a clearer separation between bay types compared to that for bacteria. The majority of the samples were clustered by geographical location (bay) or clustered by type (marina or control) for each bay pair. Only the C6/M6 overlapped by type due to one replicate.

10.1128/mSphere.00127-21.7 TABLE S4 PERMANOVA (adonis) test to assess the effect of marinas on metazoan community structure. Download Table S4, CSV file, 0.001 MB. Copyright © 2021 Iburg et al. 2021 Iburg et al. https://creativecommons.org/licenses/by/4.0/ This content is distributed under the terms of the Creative Commons Attribution 4.0 International license.

10.1128/mSphere.00127-21.8 TABLE S5 PERMANOVA (adonis) test to assess the effect of marinas on bacterial community structure. Download Table S5, CSV file, 0.001 MB. Copyright © 2021 Iburg et al. 2021 Iburg et al. https://creativecommons.org/licenses/by/4.0/ This content is distributed under the terms of the Creative Commons Attribution 4.0 International license.

### Differences in relative abundance of microbial and meiofauna taxa in marina and control bays.

**FIG 4:**
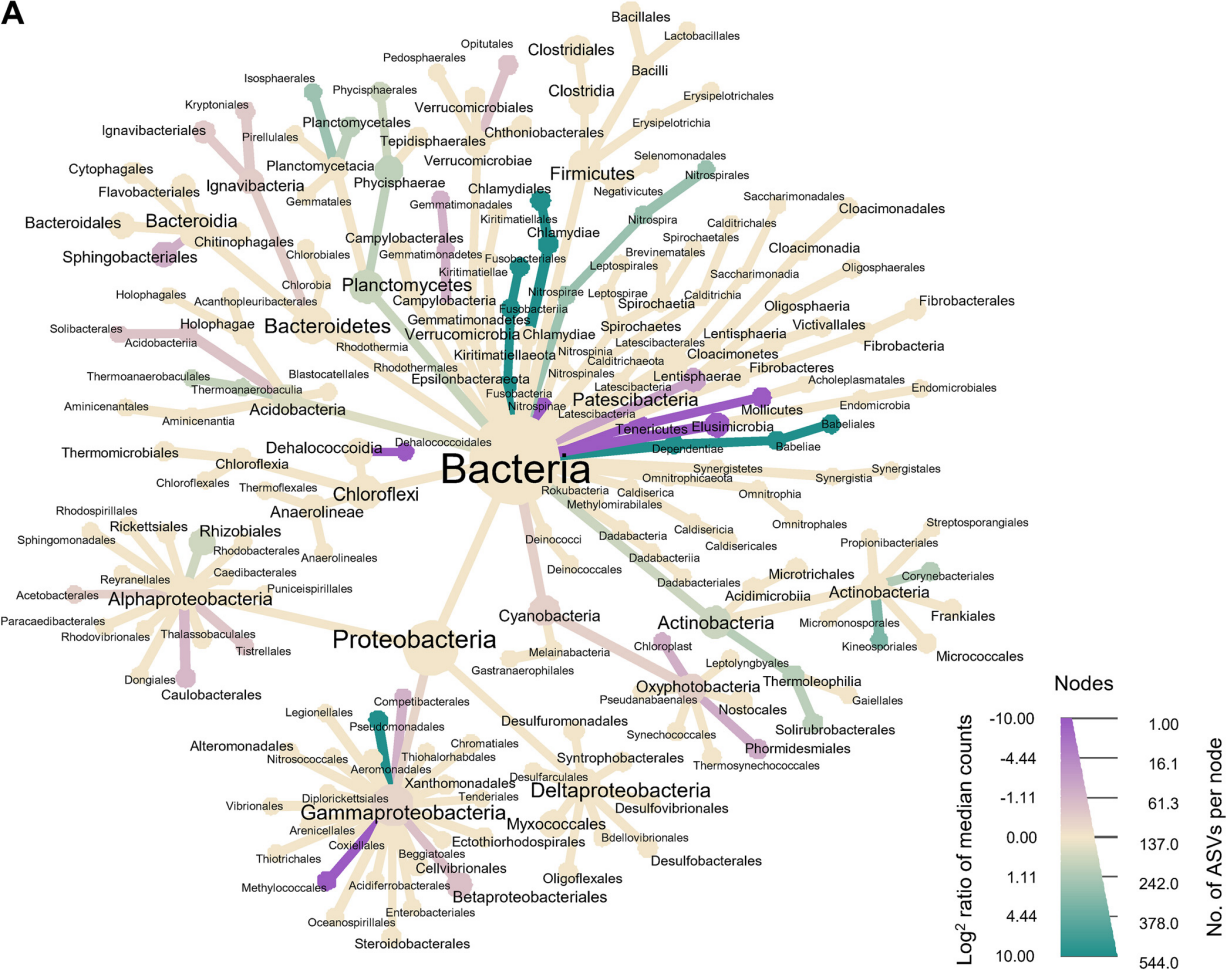

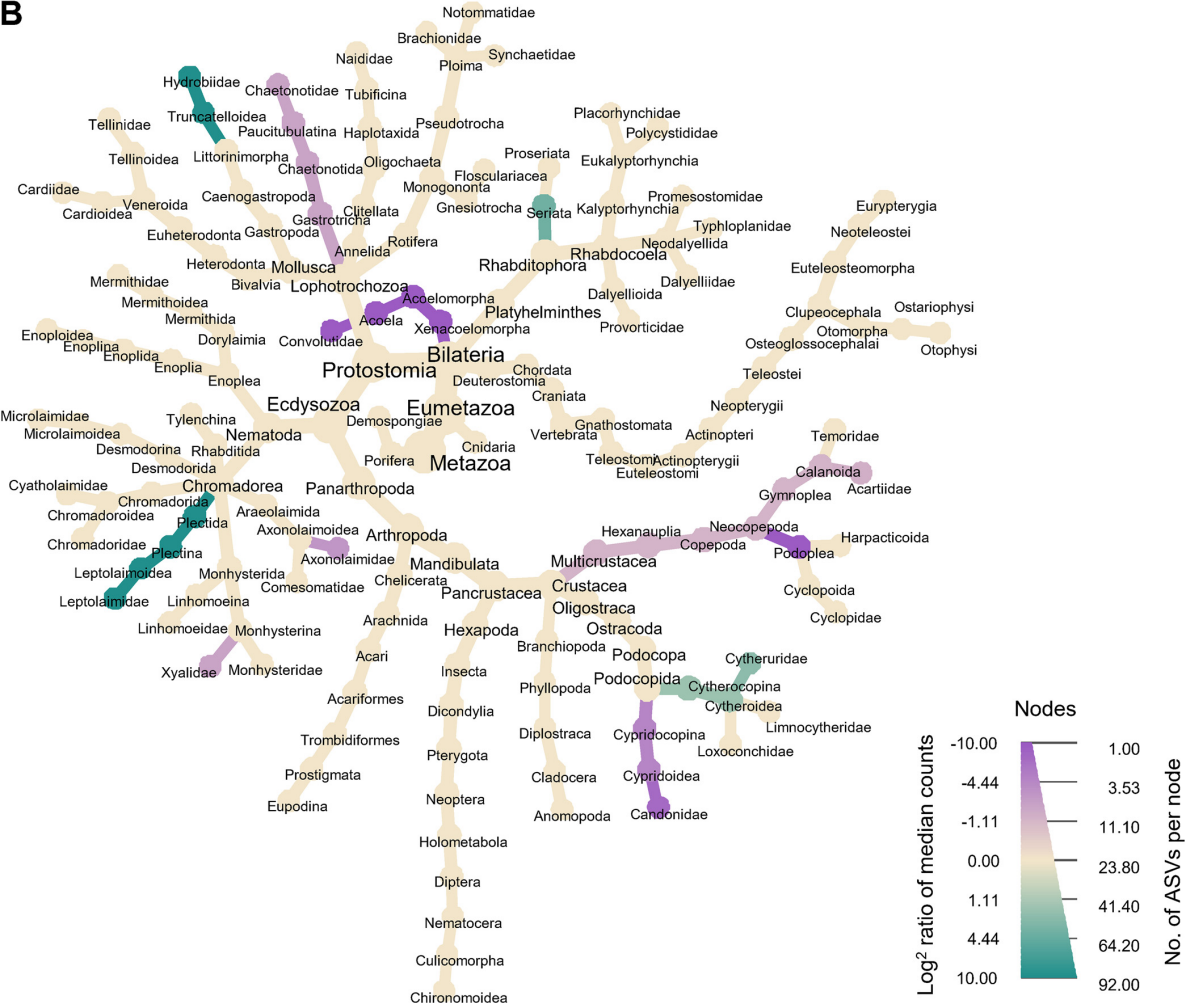
Heat tree illustrating relative abundances for microbial communities (A) (from domain/phylum to order level) and metazoan communities (B) (from phylum to family level) between control and marina bays across all bays. Nodes represent taxa used to classify ASVs, and subtaxa are depicted in their branches. The node size indicates the number of unique ASVs within that taxon, and node colors correlate with the relative abundance of organisms. The intensity of the colors is relative to the log_2_ ratio of difference in median proportions (green for control and purple for marina bays). Nodes that are colored in shades of either purple or green represent significant differences in taxon abundances, which were obtained through a Wilcox rank sum test (*P* < 0.05) followed by a Benjamini-Hochberg (FDR) correction for multiple comparisons.

Differences in relative abundances between control and marina bays were visualized by a heat tree based on log_2_ median proportions up to the order level (Fig. 4, up to the family level in Table 1), where a log_2_ median proportion of >0 indicates higher abundance in control, and a log_2_ median proportion of <0 indicates higher abundance in marinas. Relative abundance data on bacterial communities showed an increase of *Betaproteobacteriales* in marinas compared to controls (relative abundance in control: 29.18% ± 5.37%; in marina: 38.31% ± 8.82%, Wilcox *P* < 0.001, false discovery rate [FDR] corrected). At the family level, higher relative abundance of *Gallionellaceae* (log_2_ median proportion, −10), *Burkholderiaceae* (controls [C], 8.67% ± 3.87%; 12.45% ± 4.14%; log_2_ median ratio, −0.8) and *Hydrogenophilaceae* (C, 11.78% ± 7.17%; marinas [M], 15.86% ± 4.52%; log_2_ median ratio, −0.9) found in marina than in control bays (Table 1). The higher abundance of *Gallionellaceae* was based on a nearly complete absence in three of the five control bays (∼3% ± 1.3% within the order *Betaproteobacteriales*).

**TABLE 1:** Selection of bacterial and metazoan taxa of interest where relative abundances differed between control and marina bays

Organism and taxa	Relative abundance (%) (mean ± SD)^*a*^		Log_2_ median ratio
	Control bays	Marina bays	
Bacteria: class, order, and family			
*Gammaproteobacteria*			
*Betaproteobacteriales*	29.18 ± 5.37	38.31 ± 8.82	−0.72
*Burkholderiaceae*	8.67 ± 3.87	12.45 ± 4.14	−0.8
*Gallionellaceae*	0.07 ± 0.09	0.48 ± 0.43	−10
*Hydrogenophilaceae*	11.78 ± 7.17	15.86 ± 4.52	−0.9
*Methylophilaceae*	0.23 ± 0.14	0.10 ± 0.11	0
*Methylococcales*			
*Methylomonaceae*	0.14 ± 0.16	0.43 ± 0.29	−10
*Bacteroidetes*			
*Flavobacteriales*	20.75 ± 10.27	15.85 ± 4.24	
*Cryomorphaceae*	0.17 ± 0.34	0.34 ± 0.38	−10
*Crocinitomicaceae*	0.15 ± 0.19	0.64 ± 0.24	−10
*Ignavibacteriales*	12.41 ± 1.86	14.75 ± 4.75	0
*Sphingobacteriales*	0.90 ± 0.48	1.85 ± 0.30	−1.6
Meiofauna: phylum, order, class or family			
Panarthropoda	71.96 ± 10	64.67 ± 16	
Copepoda			
Podoplea	0.12 ± 0.2	0.46 ± 0.31	−1.74
Ostracoda			
Cypridoidea	1.52 ± 1.2	15.26 ± 4.8	−6.9
Cytheroidea	44.6 ± 14.9	16.03 ± 6.4	2.45
Nematoda	8.67 ± 3.9	13.1 ± 7.4	
Chromadorea			
Xyalidae	1.4 ± 2	2.69 ± 0.8	−2.12
Axonolaimoidea	1.81 ± 1	3.19 ± 4	−2.68
Leptolaimoidea	0.56 ± 0.4	0.81 ± 1	10
Xenacoelomorpha			
Acoelomorpha			
Acoela	6.90 ± 5.5	0.58 ± 0.93	−10
Mollusca			
Gastropoda			
Hydrobiidae	1.98 ± 3	0.09 ± 0.08	10
Gastrotricha			
Chaetonotida			
Chaetonotidae	1.79 ± 1.1	3.82 ± 0.7	−2.12

a The relative abundance for each type is given as a percentage (mean ± standard deviation) and amended with the log_2_ median ratios used to highlight differences in Fig. 4.

Nematoda and Panarthropoda were the dominant metazoan groups across all samples (10.36% ± 5.41% and 62.08% ± 17.10%, respectively). Looking at Nematoda, Chromadorea dominated in the C5/M5, C6/M6, and C7/M7 bays (85.36% ± 14.59%, within the Nematoda phylum). Compared by bay type, there was a slight trend to a higher relative abundance of Chromadorea in marina bays (C, 67.21% ± 25.17%; M, 70.90% ± 17.71%: Wilcox *P* = 0.08) with Axonolaimoidea driving most of the difference (C, 1.81% ± 1%; M, 3.19% ± 4%; Wilcox *P* < 0.03; log_2_ median ratio, −2.68), suggesting the difference was primarily driven by geographical location. The relative abundance of two Ostracoda families appeared to shift in opposite directions between marina and control, where Cypridoidea saw an increase in marinas (Wilcox *P* < 0.001; log_2_ median ratio, −6.9) while Cytheroidea was higher in controls (Wilcox *P* < 0.006; log_2_ median ratio, 2.45). There was a striking increase in abundance of Acoelomorpha (C, 0.58% ± 0.93%; M, 6.90% ± 5.55%, Wilcox *P* < 0.04, FDR corrected) in marina bays, except for M7 (0.05% ± 0.06%, M7 replicates, Wilcox *P* > 0.07).

### Environmental effects on community structure.

**FIG 5:**
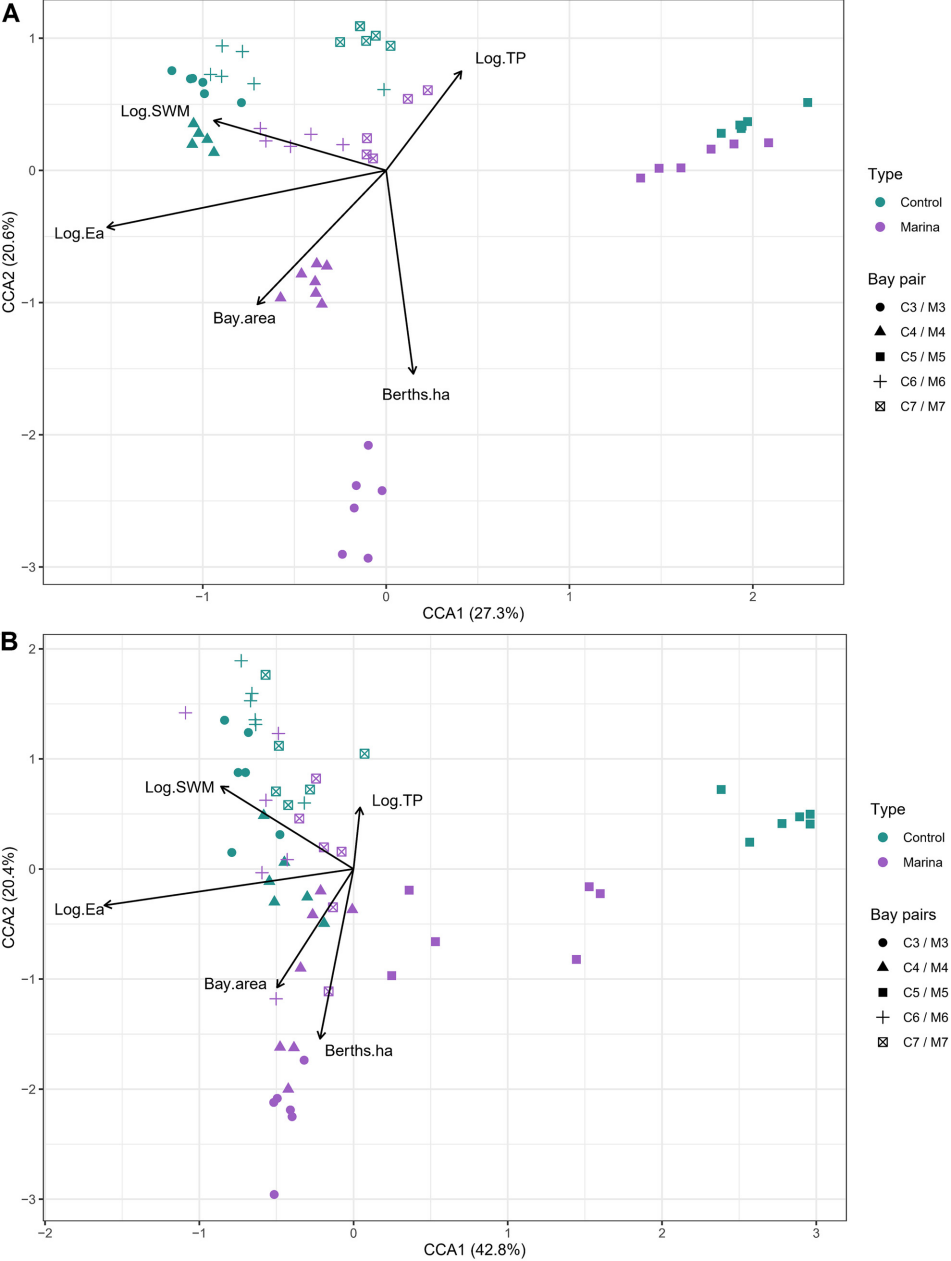
Canonical correspondence analysis (CCA) showing the relation of the bacterial (A) and metazoan (B) community structure to environmental variables. Only the variables that had significant association with the communities (*P*  ≤  0.05, Mantel test and stepwise variable selection to find the best explanatory variables) are displayed in the figure. Plotted points represent replicates for each bay and are colored by type (green for control and purple for marina bays) and shaped by bay pairs. Arrows indicate the direction and magnitude of significant variables associated with bacterial and metazoan community structure (see Table 2 and Table S6 in the supplemental material). Abbreviations: Bay.area, water surface of bay (in hectares); Log.Ea, log_10_ of simplified topographic openness; Log.SWM, log_10_ of simplified wave model (in square meters per second); Log.TP, log_10_ of total phosphorus (in micrograms per liter).

The canonical correspondence analysis (CCA) (Fig. 5) showed topographic openness (log_10_ of simplified topographic openness [Log.Ea], *F*  = 1.94, *P* = 0.005, *R*^2^adj = 0. 11), wave exposure (surface wave exposure [SWM], *F* = 1.19, *P* = 0.005, adjusted *R*^2^ [*R*^2^adj] = 0. 20), and total phosphorus (TP) (*F* = 1.18, *P* = 0.005, *R*^2^adj = 0. 25), number of berths per hectare (Berths.ha) (*F* = 0.43, *P* = 0.005, *R*^2^adj = 0. 17) to be the best predictors of bacterial community structure, explaining 47.9% of the variability. Similarly, meiofauna community composition was best explained by topographic openness (Log.Ea, *F* = 4.4, *P* = 0.005, *R*^2^adj = 0.054), water surface area of the bays (water surface of bay [Bay.area], *F* = 2.1, *P* = 0.005, *R*^2^adj = 0.071), number of berths per hectare (Berths.ha, *F* = 1.9, *P* = 0.005, *R*^2^adj = 0.085), wave exposure (log_10_ of simplified wave model [log SWM], *F* = 1.5, *P* = 0.02, *R*^2^adj = 0.093) and total phosphorous (*F* = 1.45, *P* = 0.035, *R*^2^adj = 0.1), accounting for 63.1% of the variability (Fig. 5B and Table S6).

10.1128/mSphere.00127-21.9 TABLE S6 Summary table of the stepwise selection of variables using the vegan ordistep function. Download Table S6, CSV file, 0.001 MB. Copyright © 2021 Iburg et al. 2021 Iburg et al. https://creativecommons.org/licenses/by/4.0/ This content is distributed under the terms of the Creative Commons Attribution 4.0 International license.

## DISCUSSION

In this study, we investigated the effects of recreational boating activity and other environmental predictors on micro- and meiobenthic communities. First, there was a surprisingly higher metazoan Shannon alpha diversity in marinas than control bays, suggesting a positive effect from recreational boating activity on this metric. Differences in relative abundances of several meiofauna taxa between marina and controls could be linked to lower cover of vegetation (10, 15) and higher suspended organic matter (40). Second, relative abundances of *Betaproteobacteria* were higher in marina bays, in line with previous evidence of their prevalence in ecosystems exposed to chemical pressure (41, 42). Third, compared to other variables, topographic openness appeared to be a strong predictor of both metazoan and bacterial community structure, which is in accordance with its clear effect on macrobiota in this system (43–46). Further visualization and nested analysis of the CCA data showed significant correlations in metazoan community structure compared to bacteria, with the number of berths, topographic openness, wave exposure, and total phosphorous concentration driving the differences.

### Contrasting alpha diversity between bacterial versus metazoan communities.

In contrast to our first hypothesis, high boat traffic had no significant impact on the alpha diversity of the bacterial communities. Moreover, marinas had a higher total number of metazoan taxa than the reference bays. Previous work has showed that disturbed marine vegetation habitats (10, 30), chemical pollutants and sediment resuspensions after boating events (47, 48) had a negative impact on the diversity of benthic meiofauna. For instance, the resuspension of sediment has been shown to increase benthic oxygen consumption (49), negatively affecting nutrient cycling and productivity in surface sediments and its microfauna (50, 51). Here, however, we found a higher species richness of metazoan communities in the high traffic sites (marinas than in control bays). Possibly, the “intermediate disturbance hypothesis” (52) could help explain these trends; the level of multiple stressors induced in the marinas was moderate, resulting in a reduction of the density of dominant metazoan taxa and thereby favoring the coexistence of competitive and opportunistic taxa. In fact, we found a decrease in the relative abundance of ostracods in the marina sites compared to the controls (see Fig. S2 in the supplemental material). Ostracods often dominate meiofauna biomass in the Baltic Sea sediments (53) and consume a large fraction of newly deposited organic matter (54, 55). Moreover, the ability of ostracods to acquire food resources is sensitive to changes in disturbance and intense interspecific competition (56). It is possible that similar mechanisms explain the patterns observed in our study, where a reduction in relative abundance of ostracods in marina bays may have allowed for the coexistence of a larger number of meiofaunal taxa. However, we did not detect a significant difference in meiofauna evenness between the marinas and controls. This diversity metric was on average lower in the marina bays but with high variation (Fig. S3). Furthermore, high boating activity can lead to increased input of organic matter derived from anthropogenic sources (e.g., from release of anti-fouling compounds or fuel leakage) into the sediment along with the physical damage (57, 58). Also, overall thinning of submerged macrophytes (59, 60) could create new niches for opportunistic species to colonize and possibly populate these systems (61), thereby resulting in a significant change in the alpha diversity of metazoan.

10.1128/mSphere.00127-21.1 FIG S1 Heat tree on relative metazoan abundances for control (top) and marina bays (bottom). Nodes represent taxa used to classify ASVs, and subtaxa are depicted in their branches. The intensity of the colors depict the mean relative abundance (green for control and purple for marina bays). Download FIG S1, TIF file, 1.5 MB. Copyright © 2021 Iburg et al. 2021 Iburg et al. https://creativecommons.org/licenses/by/4.0/ This content is distributed under the terms of the Creative Commons Attribution 4.0 International license.

10.1128/mSphere.00127-21.2 FIG S2 Relative abundances for select meiofauna, by bay, ordered by pairs (A) and type (marina and control) (B). Download FIG S2, TIF file, 0.8 MB. Copyright © 2021 Iburg et al. 2021 Iburg et al. https://creativecommons.org/licenses/by/4.0/ This content is distributed under the terms of the Creative Commons Attribution 4.0 International license.

10.1128/mSphere.00127-21.3 FIG S3 Species evenness for meiofauna and bacteria between control and marina bays. Download FIG S3, TIF file, 1.0 MB. Copyright © 2021 Iburg et al. 2021 Iburg et al. https://creativecommons.org/licenses/by/4.0/ This content is distributed under the terms of the Creative Commons Attribution 4.0 International license.

In contrast to metazoan overall species richness, there was no significant difference in alpha diversity of the bacteria between the marina and control bays. Considering “size-plasticity” (62) compared to meiobenthic species, bacteria are likely to be more metabolically plastic and therefore less influenced by shifting environmental conditions (37). The idea that bacteria are more plastic than metazoans when confronted with similar environmental disturbances has been supported by other studies. For example, Montagna et al. (63) demonstrated that many bacterial taxa overlapped across plant habitats subjected to different disturbance levels, while metazoa partially overlapped in the habitat experiencing the lowest disturbance level. As such, bacterial assemblages in each bay pair may be more tolerant to the levels of boat traffic studied here than metazoa.

### Bacterial and metazoan community structure.

Our second hypothesis, that community composition would differ taxonomically between high and low boat trafficked areas, was supported by our data in both bacterial and meiofauna communities. Although bacterial community structure differed between marina and control bays, it highlighted a stronger link between morphometric similarities of the bays and the benthic metazoan communities as indicated by the community relationship with environmental variables (Fig. 5). Differences in community composition between marina and control bays were more pronounced for meiofauna than bacterial communities. For meiofauna, community composition analyses showed slight clustering both by type (control/marina [Fig. 3B]) and pairs (Table 2), which was reflected in the distribution of the relative abundance among nematode classes. A clearer separation in community structure between paired bays for metazoans, than for bacteria, suggests that environmental filtering related to the boating disturbances strongly impacted metazoans (64). A constrained CCA analysis showed that topographic openness, water surface area, number of berths, and total phosphorus contributed to substantial variation in the metazoan communities between the marina versus control bays (Fig. 5A; see also Table S6 in the supplemental material).

**TABLE 2:** Environmental predictors^*a*^

Bay	Type	Pair	Veg	S.sub	Bay.area	Log.Ea	Log.SWM	Log.D	Log.TP	Log.TN	Sal	Berths.ha
C3	Control	Pair 1	116.17	5.67	5.57	0.49	3.79	1.27	−0.01	1.37	6.43	1.61
M3	Marina		46.67	3.83	8.52	0.28	3.27	1.24	−0.27	1.28	7.3	45.92
C4	Control	Pair 2	69.4	4.8	10.15	0.24	3.58	1.26	−0.26	1.28	5.7	0.3
M4	Marina		63.57	3	9.74	0.32	3.3	1.17	−0.42	1.27	5.4	16.42
C5	Control	Pair 3	63.5	2.5	3.37	−2.49	2.95	1.25	−0.24	1.44	5.3	0.59
M5	Marina		28.83	2.5	2.07	−1.54	3.14	1.12	0.24	1.5	5.5	18.84
C6	Control	Pair 4	81.83	3	1.75	−0.31	4.62	1.06	−0.1	1.35	6.35	0
M6	Marina		59	2.8	3.4	−0.3	4.63	1.12	0.02	1.33	6.1	13.25
C7	Control	Pair 5	64.33	6	3.13	−0.19	3.13	1.23	−0.05	1.34	6.6	0
M7	Marina		78	4.8	3.02	−0.35	3.06	1.15	−0.16	1.29	6.6	8.27

a Variable abbreviations: Veg, percentage of cumulative vegetation; S.sub, total number of submerged vegetation species; Bay.area, water surface of bay (hectares); Log.Ea, log_10_ of simplified topographic openness; Log.SWM, log_10_ of simplified wave model (square meter per second); Log.D, log_10_ of water depth (decimeter); Log.TP and Log.TN, log_10_ of total phosphorus and total nitrogen (micrograms per liter); Sal, salinity (practical salinity units [PSU]); Berths.ha, number of berths per water surface area of a bay (number per hectare).

In contrast to meiofauna, there was no clear trend in the community-environment relationship for bacteria. Most of the variables measured in this study were related to bay morphometry; this finding therefore further supported our early suggestion that bacteria were more plastic than metazoa when confronted with recreational boating traffic. The relationships between bacterial community and environmental conditions are likely complex and depend on the spatial scale (65). Both local scale factors (abiotic and biotic interactions) and regional scale factors (dispersal-related processes) are important determinants for a taxon’s distribution and abundance (65–67). Thousands of bacteria can be transported during boating events (68); presumably, such passive dispersal strongly influenced the assembly of bacterial communities to a greater extent than the cumulative effects of environmental factors studied here. Given the short generation time of bacteria (69), their regrowth within hours to days after environmental perturbation might have enabled them to recolonize the boating disturbed sites.

### Differences in bacterial taxonomic composition between bays.

Despite high resemblance in bacterial community composition between marinas versus controls, we identified a number of taxa whose relative abundances were significantly higher in the marinas than in the control bays. Across all marina bays, we detected an increase in the abundance of bacteria affiliated with *Hydrogenophilaceae* and *Burkholderiaceae* previously described to tolerate high concentrations of organic contaminants. Both families have been associated with hydrocarbon pollution (41, 42, 70) and may have been favored by an increase in organic pollutants released from recreational motor boats. Similarly, *Gemmatimonadetes*, while showing only a total increase of 0.11% compared to controls in our data, have been found to be present and persistent in areas contaminated with polyaromatic hydrocarbons (PAHs) (71) and in bisphenol A (BPA)-degrading sediments (72). Therefore, it is possible that these differences are related to a potential higher input of organic content associated with boating (e.g., fuel leakage, anti-fouling compounds, etc.), and/or the use by bacteria of micropollutants as carbon sources (73–76). However, such potential effects of contaminant release related to boating activities on microbial communities should be investigated in more-controlled conditions in future studies.

### Relative abundance of meiofauna in response to high boating activity.

On per-taxon abundances, there was a strikingly higher mean abundance of Acoela flatworms (Convolutidae) in marina bays and a strikingly higher mean abundance of ostracods, particularly family Cytheruridae, than in controls. These differences in relative abundances can be linked to disturbance of preferred habitats. Marina bays in our study have previously been shown to harbor less dense submerged macrophyte canopies than control bays (10). Again, increased disturbance has the potential to reduce ostracod’s capacity to acquire food resources (56) and diminishes the integrity of their preferred habitats (77, 78). However, one can expect different responses to perturbations within ostracods. For instance, Cypridoidea were found in higher abundances in marinas, while members of the same order of Podocopida, Cytheruridae, showed the opposite trend. While Cytheruridae are indicators of oligo/mesotrophic and oxygenized conditions, Cypridoidea (particularly Candonidae) has been found dominant under sediment physical disturbance (56), it is possible that the physical disturbance as a result of boating activity could favor Candonidae in marina bays. Conversely, the flatworm Acoelomorpha may be favored in marinas due to the resuspension of algae and stimulated scavenging from perturbation (53). Among nematodes, three families showed clear variations between the sampled bays: Xyalidae and Axonolaimidae abundances were found to be higher in marina sediments, whereas Leptolaimidae were notably lower than in controls. Leptolaimidae, while reported to be considerably tolerant to a variety of chemical stressors (79), has been reported to prefer vegetated sediments (80), as observed in this study. Xyalidae, however, has been linked to more unstable conditions (e.g., high hydrodynamic conditions) (81). It appears that these larger changes in diversity and composition of meiofauna in comparison to bacteria between bays with high boating versus less boating support our third hypothesis.

DNA metabarcoding has been shown to be effective for characterizing the taxonomic structure and distribution patterns of both microbial (82, 83) and (micro)eukaryotic assemblages (38, 84–86). However, this widely used approach has its limitations (87). rRNA genes are routinely used to identify the presence of micro- and macroorganisms in environmental samples, but DNA-inferred data does not distinguish between active, dormant, or dead (residual DNA) organisms. Future work using both DNA and RNA approaches would provide better insight here. Monitoring the dynamics and succession of active benthic metazoan communities could provide more accurate information to what extent the benthic organisms are impacted in ecosystems subjected to boating.

### Conclusions.

We identified several main drivers of microbial and meiofauna diversity in shallow coastal habitats of the Baltic Sea. While bays with high recreational boating showed a minor but significant difference in benthic meiofauna and microbial community structure compared to controls, other environmental predictors, like the openness, bay area, and wave exposure, were driving most of the differences. Variability in community composition of bacteria and meiofauna suggest that deterministic (i.e., environmental selection) and stochastic (i.e., passive dispersal) processes differently influenced the assembly of bacterial and meiofauna communities in bays exposed to boating disturbances. While adding to previous reports that showed negative impacts of boating activity on aquatic organisms (10, 11, 19), our results provide the first step toward understanding the responses of sediment bacteria and metazoan communities to recreational boating. This could aid in guiding management efforts aimed at protecting and managing biodiversity in coastal ecosystems and to consider overall openness and wave exposure. Considering the vital role of bacteria and micromeiofauna in benthic ecosystem function (88–92), prolonged disturbances of shallow coastal habitats that change benthic ecosystem structure are likely to impact the functioning of these ecosystems. Such impacts should be considered when managing current and future mooring developments and avoid further degradation of coastal shallow habitats.

## MATERIALS AND METHODS

### Study site and survey design.

A field survey was conducted along the Swedish coast of the central Baltic Sea in late summer (August and September) 2014, the period when aquatic vegetation reaches its maximum cover. From this survey (10), a selection of 10 coastal bays situated ∼20 km apart along an ∼200-km stretch of the central Baltic Sea was made, covering small gradients in recreational boating activity, topographic openness, and wave exposure as well as nutrient loading (Fig. 1). Five bays with high levels of boating activity were selected (hereafter “marinas”). Marinas were defined as shallow inlets with a high number (>8) of berths allocated for permanent mooring of small (below 12 m) boats in use during the boating season in Sweden (spring to autumn). The number of berths was standardized to water surface area of the bay. Marinas were paired with five “control” bays with low levels of boating activity (<2 mooring berths/ha) but matched the marina bay in its morphometry and abiotic conditions as closely as possible (10) (Table 2). The average number of berths (moorings) per bay was used as a measure of anthropogenic pressure from recreational boat traffic. The number of berths was obtained from counting during the field survey, as well as analyzing satellite images by Google Earth Pro (v 7.1.5.1557) and the Swedish mapping, cadastral and land registration authority (METRIA, Lantmäteriet; www.metria.se) for the year of field sampling (2014). All types of berths were counted, including berths on jetties, piers, docks, boathouses, and permanent mooring buoys. The number of berths approximates the actual boating pressure at the inlets, as the average boat size in the marinas was used to approximate the size of a berth when it was empty and the size was uncertain (10). Actual boat traffic was not measured but is here assumed to be closely positively related to increasing number of berths in bays.

The five marina bays were chosen to form a pressure gradient, from small boat harbors with few berths, to extensive marinas with a high number of berths. Compared to the bay selection in the field survey, with seven marina/control pairs, the two most northern marina/control pairs were less morphometrically similar and were thus excluded from this study. Within each bay, six or seven stations were sampled with one replicate per station (*n* = 61).

Sediment samples were collected between August and early September 2014. At each station, surface sediment was sampled by a snorkeler at 0.5- to 3-m water depth, using an open 20-ml syringe (2-cm diameter) to a sediment depth of 2 cm. Samples were then kept on ice and in the dark for ca. 6 h until arrival at laboratory where they were kept frozen at –20°C until DNA extraction. The percent cover of aquatic vegetation species within a 5-m radius (∼80 m^2^) was visually estimated, and the cumulative percentage cover of all vegetation (“Veg” estimates can >100%) and total number of submerged vegetation species (“S.sub,” excluding filamentous algae) was calculated. At each station, water temperature and salinity were measured using a Multi 340i voltmeter (WTW, Germany). Water samples were collected at 0.5-m depth between 4 and 7 p.m., frozen, and analyzed for total nitrogen (TN) and total phosphorus (TP) concentration measurements (in micrograms per liter) using segmented flow colorimetric analysis with the Alpkem FlowSolution IV system from OI Analytical.

Water exchange was estimated as simple topographic openness (abbreviated as *Ea*), using the formula: *Ea *= 100 × *At/a*, where *At* is the smallest cross-section area of a bay connected to the open sea and *a* is the water surface area of the bay (93). Water surface area of the bays (*a*) was estimated from satellite images in Google Earth Pro. Cross-section area (*At*) was calculated as *L* × *d*, where *L* is the length of the bay opening (measured from satellite images in Google Earth Pro) and *d* is its depth (94). The topographic openness functions as a proxy for surface-water retention time (*R*^2^ = 0.97) (93, 95), which affects abiotic variables such as water temperature, salinity, and particle sedimentation, and in turn the biological communities (43–46). As some of the surveyed bays are incorporated in an array of bays and sounds, the smallest values of *At*, facing the open sea, of these archipelago areas were used (23). Thus, the area used to calculate the water exchange may be larger than the actual area sampled, and the value of *At* facing the sea may be derived from water bodies just outside the actual bay sampled.

Surface wave exposure (here abbreviated as SWM) was estimated for each bay using the simplified wave model (96, 97). SWM (square meter per second) for a central point in each bay was calculated using a geographic information system (GIS)-based wave model, based on averages of fetch calculations from 16 compass directions with wind conditions over a 5-year period and accounting for diffraction effects (96, 97). Diffraction effects are simulated by a spreading algorithm, and fetch is an estimate of the distance over which waves can collect wind energy before reaching a site.

### Extraction of sediment eDNA.

DNA from each sample (*n* = 61; 6 or 7 samples per bay) was directly extracted from 8 to 9 g of sediment samples using DNEasy PowerMAX soil kits (Qiagen) following the provided protocol but modified to allow for four simultaneous extractions on a single vortex. The quality of extracted DNA yields was assessed using a Nanodrop spectrophotometer (Thermo Scientific) and stored at −20°C until library preparations.

### Library preparations.

Two libraries targeting the 16S and 18S rRNA genes were prepared, following the dual-index amplification methods adapted from previous work (98, 99). The hypervariable region V3-V4 of the 16S rRNA gene was amplified using the 341F/805R primer pair (82) for bacteria, and the 18S rRNA gene was amplified using the TAReuk454FWD1/TAReukREV3 primer pair (100) for eukaryotes (see Table S1 in the supplemental material). Briefly, the first round of PCR was carried out using primers amended with Illumina Adapter sequences (Table S1) to amplify the targeted 16S and 18S genes. The thermal program for both the 16S and 18S rRNA gene amplification in the first round used an initial denaturation at 98°C for 30 s, followed by 12 cycles, with 1 cycle consisting of denaturation at 98°C for 10s, annealing at 50°C for 30 s, and extension at 72°C for 30 s. All PCRs for the library preparation were carried out on a Bio-Rad T100 thermal cycler (Bio-Rad Laboratories) using Q5 HS (high-sensitivity) High-Fidelity Master Mix (New England BioLabs), following the manufacturer’s instructions. First-round amplicons were cleaned by adding 0.1 μl exonuclease I (New England BioLabs) and 0.2 μl thermosensitive alkaline phosphatase (Promega), incubating for 15 min at 37°C, followed by 15 min at 74°C to terminate the reaction. The second round of PCR was carried out using indexing primers described in previous work (98) to equip each sample with a unique combination of forward and reverse index sequences. The thermal profile for the second round was as follows: 3 min at 95°C, 15 cycles with 1 cycle consisting of 30 s at 95°C, 30 s at 55°C, and 30 s at 72°C, and a final elongation of 5 min at 72°C. The final amplification products were then cleaned using Agencourt AMPure XP magnetic beads (Beckman Coulter). The concentrations of all amplicons were measured using a Qubit 2.0 Fluorometer and the double-stranded DNA (dsDNA) BR assay kit (Invitrogen) before samples were standardized and pooled. The 2 × 300 bp paired-end sequencing was conducted on an Illumina MiSeq V3 platform at the National Genomics Infrastructure (NGI) in Stockholm, Sweden (SciLifeLab, Stockholm, Sweden).

10.1128/mSphere.00127-21.4 TABLE S1 Primers used in this study. Download Table S1, CSV file, 0.00 MB. Copyright © 2021 Iburg et al. 2021 Iburg et al. https://creativecommons.org/licenses/by/4.0/ This content is distributed under the terms of the Creative Commons Attribution 4.0 International license.

### Sequence processing.

Quality filtering and chimera removal were done using the DADA2 pipeline (101) in R (102). Forward and reverse paired-end reads for both data set (16S and 18S) were truncated and trimmed using the following parameters: truncLen = c(290,210), maxEE = c(2), trimLeft = c(8), minFoldParentOverAbundance = 4 and allowoneoff = TRUE. Taxonomic assignment for 16S rRNA amplicon sequence variants (ASVs) was carried out using the SILVA database (r.132) (103) and the DECIPHER package (v 2.10.2) (104). Singletons (i.e., ASVs occurring only once across samples) were removed from both data set. After taxonomy was assigned, the relative abundance of each taxon was calculated from the proportion of that taxon relation to a total count of a particular sample.

For eukaryotic ASVs, sequences were aligned against the National Center for Biotechnology Information (NCBI) NT database using BLAST. The output file was imported to MEGAN (v 6.14.2) (105), and hits for NCBI NT association numbers were linked to taxonomic classifications. Sequences affiliated with Metazoa in the taxonomic description were extracted from the 18S data set and analyzed further as relative abundances. The raw sequences/data set can be found on the NCBI repository (BioProject accession no. PRJNA694832).

### Data analyses.

Alpha diversity of the 18S rRNA and 16S rRNA data sets was obtained as observed richness (total count of ASVs observed), Chao1 (estimator of species richness based on abundance), and Shannon diversity (estimator of both species richness and evenness) (106) as implemented in the phyloseq package (v1.24.2) (107). A dissimilarity matrix was generated on data sets using the proportional read counts with Bray-Curtis distance, which was then used to create a nonmetric multidimensional scaling (NMDS) ordination plot.

As meiofauna are known to respond quickly to physical and chemical disturbance (108, 109) and to degradation of macrophytes (30), we directed our focus to bacteria and meiofauna by filtering for metazoans in the 18S data set. For both bacterial and meiofauna communities, differences in alpha diversity between marinas and control bays were investigated with mixed effects models using the *lme4* package (110). Marina/control type was set as a fixed factor, and bays nested in marina-control pairs were set as random factor, using stations as replicates. *P* values were calculated using the *lmerTest* package (111) and the Satterthwaite’s approximation for denominator degrees of freedom. Pseudo-*R*^2^ was parsed into variance related to the fixed factors (marginal *R*^2^) and variance related to the fixed and random factors (conditional *R*^2^) using the *MuMIn* package (112).

To visualize differences in metazoan and bacterial relative abundance between marina and control bays, an NMDS ordination plot using Bray-Curtis distance dissimilarity matrix. This was complemented by permutational multivariate analysis of variance (PERMANOVA), carried out to assess the effect of bay types on the bacterial and meiofauna community structures, we used type (two levels, control and marina) as a fixed factor and pair as a nesting variable with the *adonis* function in the *vegan* package (113).

Differences in the relative abundances of taxa between marina and control bays were analyzed for phylum, class, and order levels separately using the proportional read counts with the *phyloseq* (107) and *metacoder* packages (114). A heat tree was used to illustrate taxonomic affiliation and the degree of abundance difference. Within the heat trees, differences in relative abundance of taxa between control and marina bays were obtained from log_2_ median proportion values, and significant differences were tested with a Wilcox test using the *vegan* package and highlighted after false discovery rate (FDR) corrections.

Multivariate relationships between the measured environmental predictors (Table 2) and meiofauna and bacterial community composition were explored using canonical correspondence analysis (CCA) (115) with the cca function from the *vegan* package (113), while constraining the permutation residuals of the bacterial and meiofauna community data to comparisons between pairs of marina and control bays. Community compositions were calculated using the Bray-Curtis dissimilarity on square root-transformed relative abundances, while environmental predictors were log transformed before analysis. Environmental predictors significantly affecting bacterial and meiofauna community composition were first selected based on forward selection with the function *ordistep* in *vegan*. We then used an analysis of variance (ANOVA) to assess the significance of constraints with a linear model. Only significant variables (*P* < 0.05) were included in the CCA triplots.

### Data availability.

The FASTQ files and associated metadata are publicly available at the National Center for Biotechnology Information under the accession number PRJNA694832.
