# Supplementary figures and images for: Effects of Recreational Boating on Microbial and Meiofauna Diversity in Coastal Shallow Ecosystems of the Baltic Sea
Source: mSphere. 2021 Sep 1;6(5):e00127-21. doi: 10.1128/mSphere.00127-21 (PMC8550262; doi:10.1128/mSphere.00127-21)

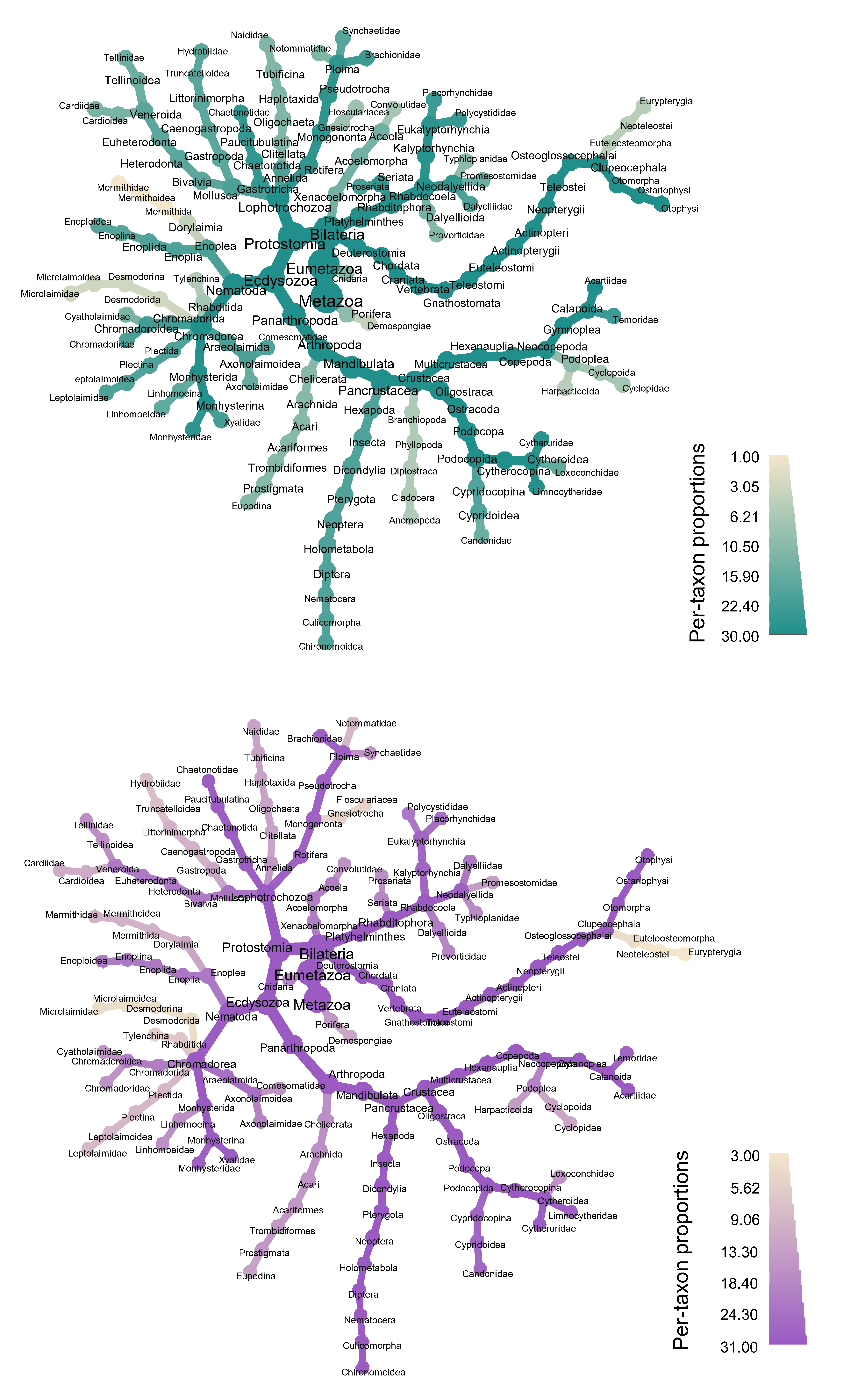

Supplement: FIG S1 [file msphere.00127-21-sf001.tif]

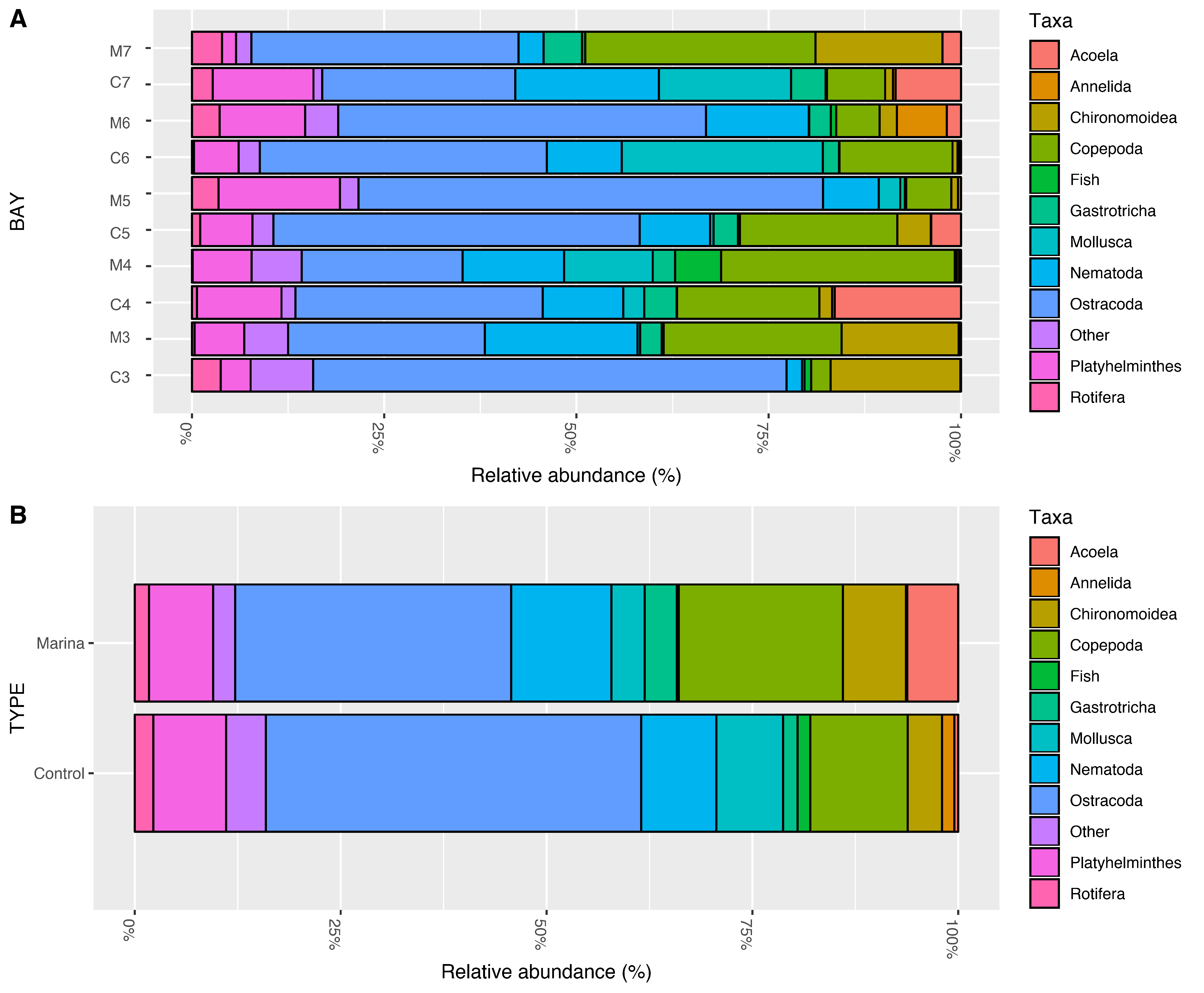

Supplement: FIG S2 [file msphere.00127-21-sf002.tif]

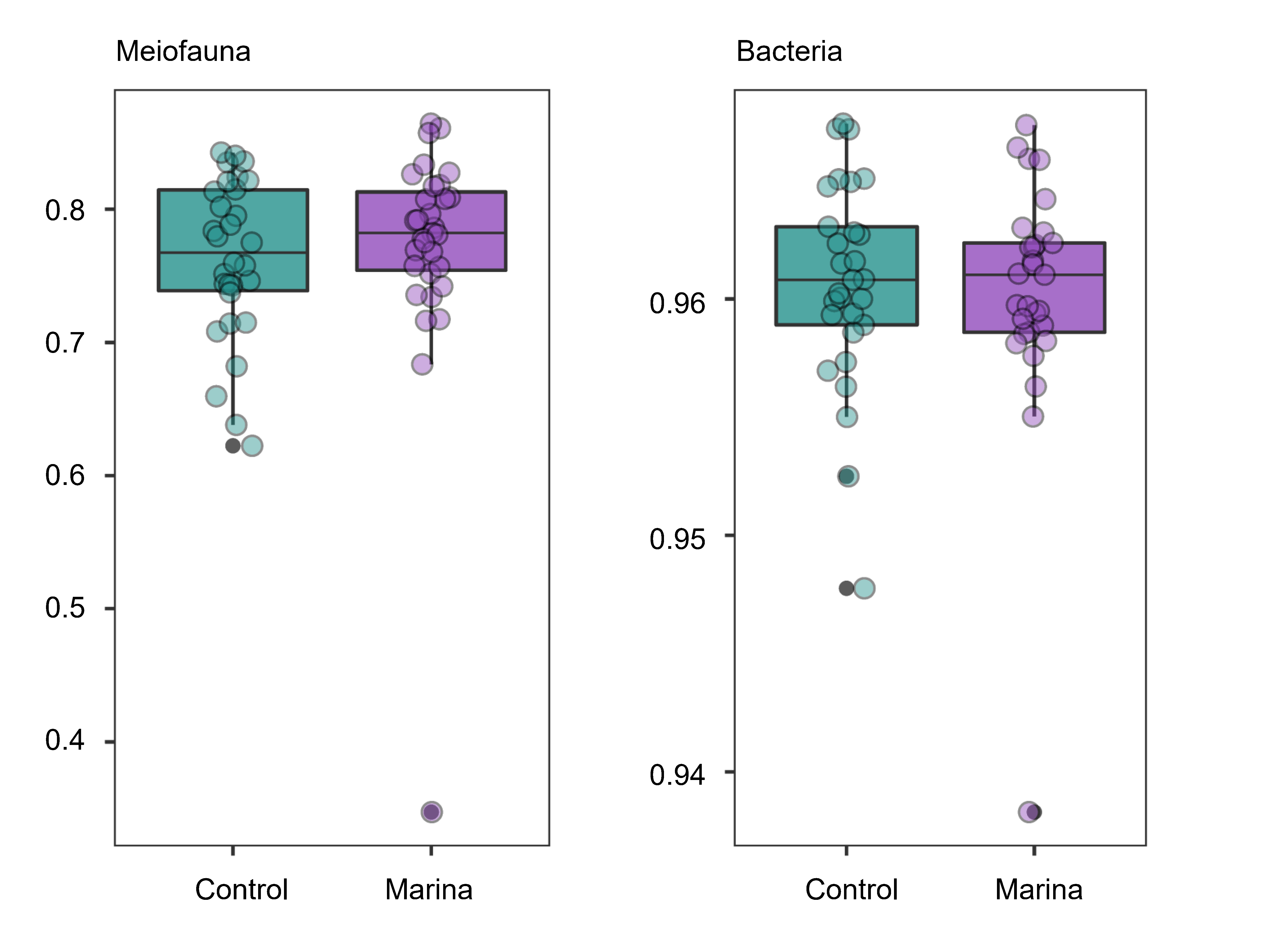

Supplement: FIG S3 [file msphere.00127-21-sf003.tif]
